# Travel routes to remote ocean targets reveal the map sense resolution for a marine migrant

**DOI:** 10.1098/rsif.2021.0859

**Published:** 2022-05-11

**Authors:** Graeme C. Hays, Nadine Atchison-Balmond, Giulia Cerritelli, Jacques-Olivier Laloë, Paolo Luschi, Jeanne A. Mortimer, Alex Rattray, Nicole Esteban

**Affiliations:** ^1^ Deakin University, Geelong, Victoria, Australia; ^2^ British Indian Ocean Territory, King Charles Street, London SW1A 2AH, UK; ^3^ Department of Biology, University of Pisa, Via A. Volta 6, 56126 Pisa, Italy; ^4^ Department of Biology, University of Florida, Gainesville, FL 32611, USA; ^5^ PO Box 1443, Victoria, Mahé, Seychelles; ^6^ Department of Biosciences, Swansea University, Swansea SA2 8PP, UK

**Keywords:** navigation, route-finding, current drift, BIOT, Diego Garcia

## Abstract

How animals navigate across the ocean to isolated targets remains perplexing greater than 150 years since this question was considered by Charles Darwin. To help solve this long-standing enigma, we considered the likely resolution of any map sense used in migration, based on the navigational performance across different scales (tens to thousands of kilometres). We assessed navigational performance using a unique high-resolution Fastloc-GPS tracking dataset for post-breeding hawksbill turtles (*Eretmochelys imbricata*) migrating relatively short distances to remote, isolated targets on submerged banks in the Indian Ocean. Individuals often followed circuitous paths (mean straightness index = 0.54, range 0.14–0.93, s.d. = 0.23, *n* = 22), when migrating short distances (mean beeline distance to target = 106 km, range 68.7–178.2 km). For example, one turtle travelled 1306.2 km when the beeline distance to the target was only 176.4 km. When off the beeline to their target, turtles sometimes corrected their course both in the open ocean and when encountering shallow water. Our results provide compelling evidence that hawksbill turtles only have a relatively crude map sense in the open ocean. The existence of widespread foraging and breeding areas on isolated oceanic sites points to target searching in the final stages of migration being common in sea turtles.

## 1. Introduction

Understanding how animals navigate in the open ocean is a long-standing question, despite many tens of thousands of fish, mammals, birds and turtles being tracked [1]. On land, animals are often moving through an environment rich in potential navigational information [2] and they may use a variety of cues to help direct their movements including scent trails, visual landmarks and olfactory cues [3]. By contrast, in the open ocean far from land, animals may have more limited information and so face especially challenging navigational tasks, particularly when they are trying to locate specific isolated targets [4,5]. For sea turtles, fish, seabirds and marine mammals, repeated migrations often take place between individually specific breeding and foraging areas. How these migrations are completed has perplexed biologists, including Charles Darwin, for more than a century [6] and remains a key unresolved question, with sea turtles often considered iconic oceanic migrants because of the huge distances they migrate, often to small, isolated targets [5].

Migrating animals may face various navigational tasks, each having associated cognitive requirements [7]. For example, 20 years ago elegant laboratory experiments provided a breakthrough showing that sea turtles aiming to travel to a specific destination are able to perceive components of the Earth's magnetic field that may provide a map sense in the open ocean [8]. While field studies have provided support for geomagnetic navigation in sea turtles [4,9] and similar findings have been obtained for other long-distance navigators [10,11], it remains uncertain whether this map sense is highly accurate, allowing precise pinpoint migration to isolated targets, or instead provides a cruder map that enables animals to stay roughly on the correct path [12]. In some cases, a crude map may be all that is needed, for example to direct post-hatchling turtles living in the open ocean to swim broadly north, south, east or west to find suitable areas [13]. Similarly, adults migrating to a mainland target to forage or breed may simply need an approximate heading to follow and then can correct their course when they encounter land [14–16]. However, a crude map alone may not be sufficient to locate specific targets such as remote isolated islands or submerged banks. Some animals likely have a good memory of migration routes they previously completed and routes may also be influenced by parents and other conspecifics, such as in some terrestrial birds [7]. However, for animals travelling through the open ocean far from any visible land or bathymetric features, memory likely plays less of a role.

Theoretical considerations suggest that a geomagnetic map will not allow precise homing to isolated targets in the open ocean [17,18]. However, empirical evidence from free-living individuals for the resolution of any navigational map remains scant and obtaining this evidence is often hampered by the fact that the target of oceanic movements is generally not known for many animals, such as pelagic fish, despite large numbers being tracked. In this regard, aspects of the life history of hard-shelled sea turtles make them a good model species for assessing navigational performance. While adults from the same breeding site may disperse widely at the end of the nesting season, each individual travels to a particular foraging site to which it generally maintains fidelity throughout its adult life [19]. Adult green turtles (*Chelonia mydas*) and hawksbills (*Eretmochelys imbricata*) both primarily forage at benthic habitats, respectively feeding predominantly on seagrass and macro-algae and on sponges [20,21]. So when crossing the open ocean, they likely cannot forage en route and experience substantially reduced forage availability anywhere but at their target, or when passing over shallow waters. Therefore, the routes that these sea turtles follow to isolated targets may shed light on the accuracy of their navigational mechanisms [5]. When migrating long distances to isolated targets green turtles often show meaningful course corrections en route but then also protracted searching behaviour in the final stages of migration [15]. These recent findings suggest that navigational maps in sea turtles may be insufficiently detailed to allow pinpoint homing.

Building on these previous findings, here we examine two alternative hypotheses. If turtles have a large-scale, high-resolution map sense, they might be expected to generally show pinpoint migration to isolated targets, regardless of the distance to the target and so the route straightness index (that is, the ratio of the beeline distance between breeding and foraging site to the length of migration track) will be high regardless of migration distance. Alternatively, if turtles have a coarse-scale map then we can envisage that all migrations to isolated targets, regardless of their straight-line distance, will often end with a transition to target searching, once turtles are closer to the target than their map resolution. According to this scenario, even relatively short migrations will still be challenging and may require target search and have a low straightness index. Here, we consider these alternative hypotheses using a unique high-resolution tracking dataset for hawksbill turtles migrating relatively short distances in a central ocean area, and in this way, we provide some of the strongest evidence to date for the general processes by which sea turtles likely locate targets.

## 2. Material and methods

### 2.1. Turtle tracking

While they were ashore nesting on the island of Diego Garcia in the Chagos Archipelago, Indian Ocean (7.428° S, 72.458° E) during 2018 and 2019, hawksbill turtles were equipped with Fastloc-GPS Argos satellite tags (SPLASH10-BF, Wildlife Computers, Seattle, Washington, USA). Transmitters relayed data via the Argos system (http://www.argos-system.org/) that allowed Fastloc-GPS positions to be determined. Only Fastloc-GPS positions obtained with a minimum of four satellites and a residual error value (a dimensionless unit produced in Fastloc-GPS processing) of less than 35 were used, producing locations that were generally within a few tens of metres of the true location [22]. We identified when turtles arrived at their foraging grounds, as indicated by individuals travelling to localized, relatively shallow areas where they remained for several months before tags failed [15].

### 2.2. Ocean current and turtle tracking analysis

Migration trajectories between nesting beaches at Diego Garcia atoll and submerged banks in the Chagos Archipelago were linearly interpolated from Fastloc-GPS positions to provide locations at 6 h intervals for each turtle migration track. For each interpolated location, we associated the sea currents encountered from the 6 h Copernicus GLOBAL Ocean Sea Physical Analysis and Forecasting models, which has a spatial resolution of 0.08° (about 8.9 km at these latitudes). The sea current flows are modelled at varying depths and, in our case, we chose to use the data from a depth of 1.5 m given that turtles generally migrate remaining in the upper layers of the water column. The linear interpolation of the turtle tracking data was performed so that we had sections of track corresponding with the 6 h resolution of the ocean current information. The mean number of daily Fastloc-GPS locations during migration was 7.05 (s.d. = 2.33), and the interpolated tracks were very similar to the real track. Migration and beeline distances were calculated using the Vincenty formula in the R package ‘Geosphere’ (version 1.5-10) [23] on the WGS-84 (World Geodetic System 1984) ellipsoid. Submerged banks were defined by the 200 m isobath obtained from the General Bathymetric Chart of the Oceans 15 arc-second interval (approximately 450 m) grid. Speed of travel and heading over the ground between successive interpolated locations of migration tracks were calculated following the shortest geodesic path using the R package ‘move’ (v. 4.0.6) [24]. Departure directions of turtles from Diego Garcia were estimated for Fastloc-GPS locations obtained after at least 5 km of travel since turtles left the island on their post-nesting migration. Departure heading, headings during migration and circular statistics were derived using the ‘circular’ package (v. 0.4-93) [25] in R software v. 4.1.1 [26]. We defined the beeline to the foraging target as the shortest migration possible and then calculated the straightness index of tracks as this beeline distance divided by the total distance travelled [15]. We also calculated the perpendicular displacement from the beeline when each turtle crossed the 200 m isobath of the Great Chagos Bank after their oceanic crossing. The observed ground-related track of turtles is due to both current advection and turtle active swimming. We subtracted the current velocity (speed and direction) from each 6 h ground-related travel vector, to calculate the turtles' active swimming vectors [15]. We excluded from the analysis the tracks from 2 of the 17 turtles that migrated from Diego Garcia to the Great Chagos Bank because current data were not available on these occasions from the Copernicus GLOBAL Ocean Sea Physical Analysis and Forecasting models.

### 2.3. Comparison with other studies

To examine if the results from hawksbill turtles were consistent with those recently reported for green turtles in the western Indian Ocean, we used green turtle data from [15] on the straightness index versus beeline distances to the target.

## 3. Results

### 3.1. Turtle ground-related tracks

**Figure 1.**
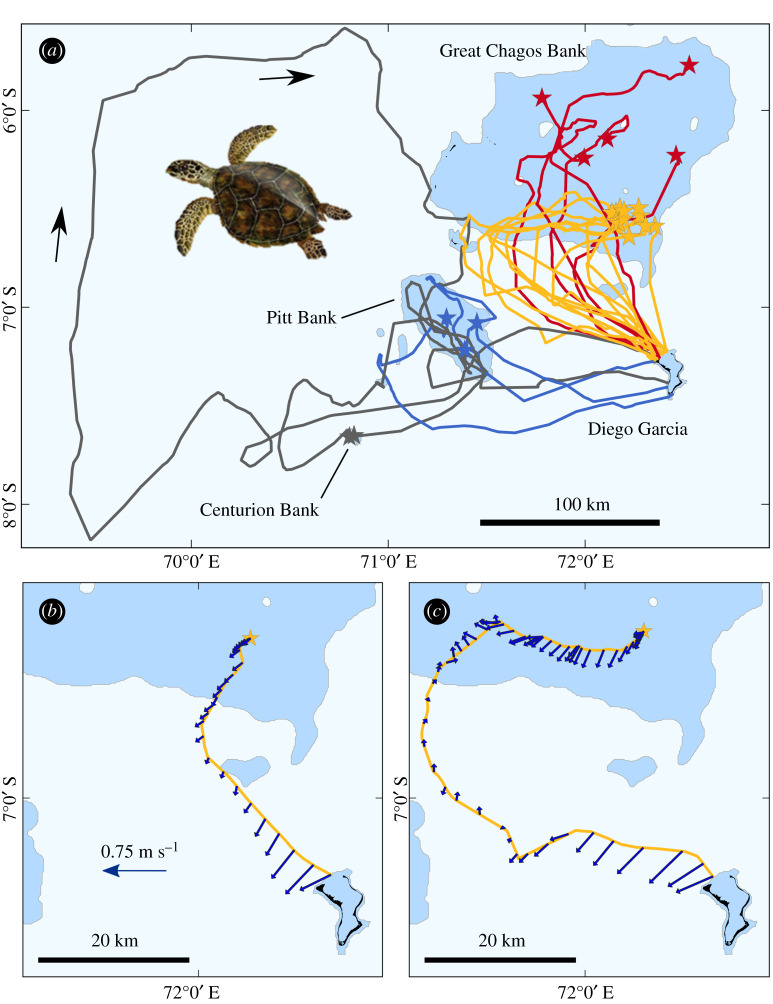
(*a*) The tracks of 22 hawksbill turtles from their nesting beaches on Diego Garcia island, to their foraging sites on the Great Chagos Bank, Pitt Bank and Centurion Bank. Stars denote the foraging site. Colours are simply to aid clarity and reflect the target, i.e. the foraging site of each turtle, with red and yellow indicating north and south of the Great Chagos Bank, dark blue Pitt Bank and grey Centurion Bank. Banks with water depths shallower than 200 m are shaded in darker blue. Arrows indicate the direction of travel for the most circuitous route. (*b*,*c*) Current vectors are shown for two examples of turtles migrating to the Great Chagos Bank. The current vectors are shown every 6 h throughout each track and so the vectors appear closer to each other when the turtle is travelling slower.

In total, 22 post-nesting hawksbill turtles were tracked from Diego Garcia Island to their distant foraging sites (figure 1; electronic supplementary material, table S1). All 22 individuals migrated to foraging sites that were on submerged banks within the Chagos Archipelago: 17 on the Great Chagos Bank, 3 on the Pitt Bank and 2 on the Centurion Bank. The beeline distance to the foraging sites was always less than 200 km (mean 106.0 km, range 68.7–178.2 km), but despite these relatively short distances, often turtles made circuitous trips to arrive at these destinations. The mean straightness index of these tracks to the foraging grounds was 0.54 (range 0.14–0.93, s.d. = 0.23). In other words, turtles typically travelled twice (and in one case seven times) the direct distance to their target. For example, one individual travelled 1306.2 km when the beeline to the foraging site was only 176.4 km. While some turtles followed fairly direct routes to the target, examples of these tracks were rare, with only 4 of 22 routes having a straightness index greater than 0.9.

**Figure 2.**
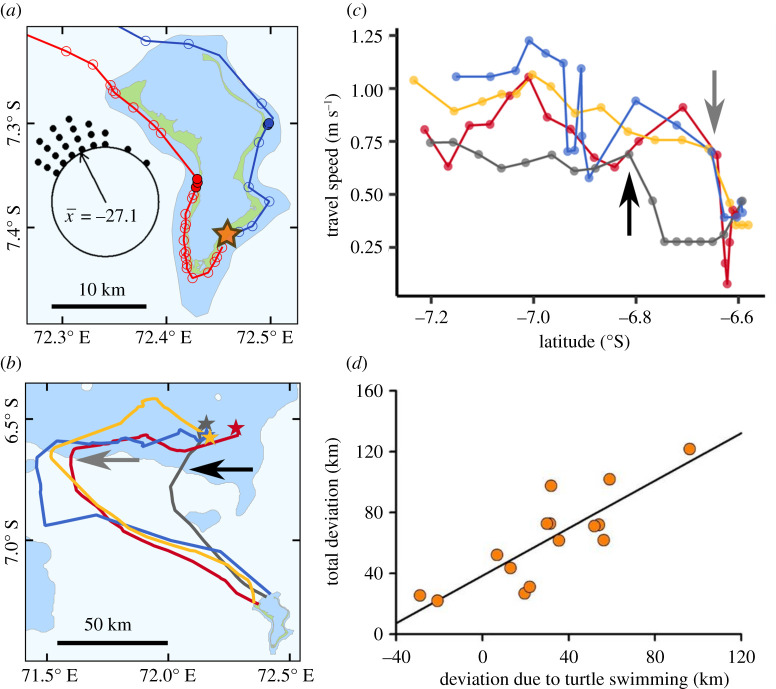
(*a*) The initial tracks of two hawksbill turtles beginning their post-nesting migration (open and filled symbols represent daytime and night-time locations, respectively). Water depths shallower than 200 m are indicated by blue shading. On departure from nesting beaches at the southeast of Diego Garcia island (orange star), turtles moved to the northern end of the island without any noticeable delay only slowing down at night. Inset shows the initial departure directions of 22 hawksbill turtles from Diego Garcia. The direction of the beeline route to the target is represented by 0° with the departure angle plotted in 10° increments. Turtles tended to head westwards of the direct route to their target. (*b*) Turtles tended to initially follow very straight-line oceanic crossings and then often turned to a more target-oriented direction when either in the open ocean or when they encountered shallow water. In this case, four tracks are shown for hawksbills migrating to the foraging sites on the Great Chagos Bank. (*c*) Associated with the reorientation in shallow water, the speed of travel tended to slow. Arrows in (*b*) and (*c*) indicate when two individuals encountered shallow water (less than 200 m). (*d*) For turtles migrating to foraging sites on the Great Chagos Bank, the total deviation off the beeline to the target after they completed their oceanic crossing versus the amount of this deviation that was caused by the turtles' active swimming. Negative values indicate that the turtles’ active swimming helped compensate for current advection, which only occurred in 2 of 15 cases. Total deviation (km) = 38.4 + 0.781 (deviation due to swimming; *r*^2^ = 0.69, *F*_1,13_ = 28.9, *p* < 0.001).

Individuals initially travelled along the coast from their nesting sites on the southeast coast of Diego Garcia to their departure points in the northwest (figure 2*a*). Turtles tended to move slowly along the coast, generally travelling between 0.1 and 0.6 m s^−1^ and did not remain stationary for long periods (e.g. days) prior to departing on their oceanic migration. The mean period between departure from nesting beaches and the start of the oceanic crossing, which was defined as the first Fastloc-GPS location in water deeper than 200 m, was 37.2 h (range = 5.02–71.98 h, s.d. = 16.2 h). Turtles generally left Diego Garcia shortly after dawn on the day of departure (mean = 4.6 h after nautical dawn, range = 0.02–15.9 h, s.d. = 4.04 h). Initial departure directions from Diego Garcia were only imprecisely target oriented, with mean of the deviation of the departure direction from the direction to the goal being −27.1° (range = −65.4 to +54.0°) (figure 2*a*). Once turtles had departed from Diego Garcia, they tended to follow straight-line courses during the initial ocean crossings, with the mean straightness index for these ocean legs of the migration being 0.91 (range = 0.66–0.99, s.d. = 0.09).

Often in the open ocean or upon arriving at the Great Chagos Bank, turtles that were off the beeline tended to reorient towards the target, with tracks changing from a more northwesterly direction to a more northnortheasterly direction (figures 1 and 2*b*). When turtles arrived in shallow water they also tended to slow down (figure 2*b,c*). For example, during migration, the mean speed of travel when water depth was deeper than 200 m and shallower than 200 m was 0.64 m s^−1^ and 0.22 m s^−1^, respectively (*t*_21_ = 14.74, *p* < 0.001).

### 3.2. Impact of ocean currents

Ocean currents were generally flowing to the southwest during the turtles' oceanic crossings (electronic supplementary material, movie SM1). The mean current speed encountered by turtles on their oceanic crossing from Diego Garcia to the Great Chagos Bank (*n* = 15) was 0.29 m s^−1^ (range = 0.11–0.39 m s^−1^, s.d. = 0.08 m s^−1^), which was less than half of the turtles’ mean active swimming speed of 0.73 m s^−1^ (range = 0.52–0.88 m s^−1^, s.d. = 0.12 m s^−1^). The mean current direction encountered by these turtles travelling to the Great Chagos Bank was 227.8°. Any lateral displacement of the ground track off the beeline to the target will be the result of current advection and/or turtle active swimming. We partitioned these two effects for turtles migrating to the Great Chagos Bank by summing the magnitude of (i) the current advection and (ii) the turtle active swimming in the direction perpendicular to the beeline to the goal. In this way, we assessed how much of the movement over the ground perpendicular to the beeline was caused by (i) and (ii), respectively. When these turtles encountered shallow (less than 200 m) water on the Great Chagos Bank, their mean distance off the beeline to target was 62.22 km (range 22.31–121.57 km, *n* = 15). The turtles' active swimming explained the majority of the total deviation off the beeline, rather than current advection (figure 2*d*). There was no evidence that the turtles corrected for current drift, with the turtles’ active swimming only reducing the deviation off the beeline in 2 of the 15 cases compared to the deviation caused by the ocean currents alone.

### 3.3. Comparison with longer migrations

**Figure 3.**
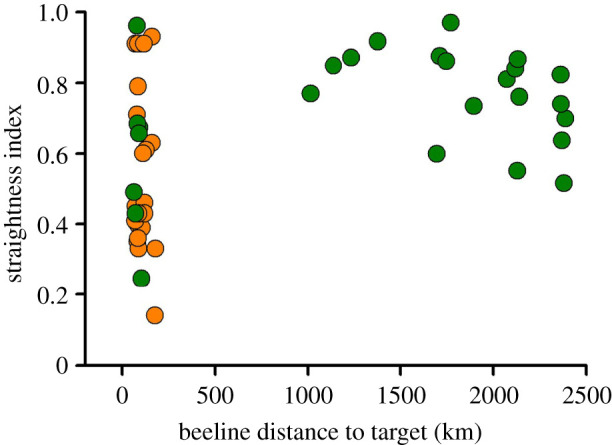
For hawksbill (*n* = 22, brown circles) and green (*n* = 26, green circles) turtles migrating from Diego Garcia to remote oceanic islands and submerged banks, the straightness index of migration routes versus the direct distance to the target. Shorter migrations tended to have lower straightness index values. For example, when the beeline distance to the target was less than 200 km versus greater than 200 km, the mean straightness index values were 0.55 and 0.77, respectively. When the beeline to the target was less than 200 km, there was no significant difference between the straightness index of green versus hawksbill turtles (*t*_10_ = 0.52, *p* > 0.05).

Like hawksbill turtles, some green turtles nesting on Diego Garcia have been tracked migrating to the Great Chagos Bank (*n* = 7), but many have been tracked migrating to distant foraging sites on oceanic islands and submerged banks in the Indian Ocean (*n* = 19). For hawksbills, the track straightness index was not related to the beeline distance to the target (*F*_1,20_ = 0.2, *p* > 0.05). The short-distance migration subsample of green turtles closely resembled that of the hawksbills, with a broad range of straightness index values (figure 3). When viewed together, for these green and hawksbill turtle migrations, the mean straightness index was lower when the beeline distance to the target was less than 200 km (mean straightness index = 0.55, *n* = 29, s.d. = 0.23) compared to when it was greater than 200 km (mean straightness index = 0.77, *n* = 19, s.d. = 0.13) (*t*_45_ = 4.33, *p* < 0.001). In other words, green turtles travelling to more distant targets tended to have more direct routes than green turtles and hawksbill turtles travelling to closer targets (figure 3).

## 4. Discussion

Our findings suggest that sea turtle navigational abilities are far from perfect, but rather may simply be as good as possible within the constraints of their sensory ability. It is often implied that migration to distant targets will present more challenges than more local migrations but the influence of migration distance on behavioural variation is seldom examined [27]. Our findings clearly show that even relatively short ocean migrations to isolated targets can present major challenges and point to turtles not using a high-resolution navigational map. Our results show the similar navigational performance of hawksbill and green turtles migrating short distances to targets. These findings are important as they reveal how across turtle species, a navigational map, for example, the geomagnetic map which has been inferred through laboratory experiments with juvenile turtles [8], does not allow pinpoint homing to isolated targets. Rather it appears that turtles have a crude map with a resolution of many tens or even a few hundred kilometres and often it is only when they are well off track that they reorient (this study) [15].

Ocean currents do not affect our conclusions in that currents may simply move turtles to new areas from where they have to navigate using their map sense. Protracted search for isolated targets by sea turtles contrasts with findings for sea birds travelling to isolated islands, where their fast speed of travel and use of wind-borne olfactory cues to navigate means that any search during the final stage of migration is generally completed quickly [28]. While there is some evidence that turtles may use wind-borne, likely olfactory, cues for island detection in special situations where the wind direction is very constant (e.g. [29]), a land-smell will not be available from a submerged bank target, which may further increase the navigational difficulty of finding such targets [15]. Furthermore, their intermittent surfacing and slow speeds of travel mean that detecting gradients in any olfactory cue is likely more challenging for a sea turtle than a flying bird.

Since turtles show tight fidelity to individual foraging sites [19], our tracked individuals likely left their foraging site targets several months previously, when they departed on their migration to breed at Diego Garcia. So individuals have almost certainly visited their migration target before and have some knowledge of the local shallow water area around this target. There are other possibilities beyond the two hypotheses we considered for how migrations are completed. For example, another possibility is that animals have a high-resolution map over a ‘home’ area they have visited previously and little or no ability to assess where they are outside this ‘home’. However, this idea is inconsistent with long-distance tracks that show turtles reorienting in the open ocean so that they arrive in the vicinity of their ‘home’ before localized searches [15]. Another plausible model is that turtles maintain a fixed direction when swimming across the open ocean and so will tend to miss the target by a greater distance on longer migrations. However, some evidence suggests this is not the case. For example, for green turtle migrations, the routes followed are not consistent with a fixed swim direction, but rather show course corrections en route [15]. Similarly, it appeared that hawksbill turtles sometimes corrected their course in the open ocean, before reaching submerged banks, when they were still in very deep water (many hundreds of metres). This finding is consistent with mid-course navigational corrections reliant on a course map sense, possibly magnetic, as has been suggested by sensory trials with captive juvenile turtles [13]. We appreciate that the straightness index may not fully capture path sinuosity and alternative approaches to track analysis may lead to further insights [30].

For some birds and insects, the timing of migration is often delayed until favourable wind conditions occur, so that the energetic costs of migrations are reduced [31–34]. However, hawksbill turtles simply departed from their island-nesting area without delay, as is commonly found in turtles tracked during their post-nesting migrations (e.g. [15,35]). These contrasting findings are likely due to the fact that the timing of turtle post-nesting migrations is largely determined by their having completed the egg-laying cycle, as is also the case in some birds and insects. However, one additional factor may be that birds and insects can perceive the wind direction while they wait to begin migration, sometimes likely by sampling the winds by making an ascent before deciding to initiate migration, but while turtles are close to shore they likely cannot perceive the oceanic currents they will encounter en route. So it appears that turtles do not optimize their migrations with respect to the most favourable ocean currents [36].

Turtles travelling to targets on mainland coasts may simply have to make the correct decision to turn left or right when reaching the coast and then can follow the shore to their destination [14,37]. In a similar way, migrating birds may reorientate homewards when they reach land after a sea crossing [28] and many terrestrial animals use visual landmarks to orientate [38,39]. Green turtles [5,15] and hawksbill turtles (this study) may reorientate in the open ocean when off the beeline to their target, suggesting a crude map sense. Additionally, even though the tracked hawksbill turtles did not encounter any land during their migrations, they often appeared to derive navigational information when they reached shallow water, as evidenced often by a clear reorientation towards their target. In shallow water, turtles can see the bottom and so they may switch to using landscape features. The navigational information might take the form of a cognitive map of shallow areas that turtles have visited previously. Alternatively or additionally, encountering shallow water may make turtles re-assess their position, rather than simply following the same course that they have maintained in the open ocean, so that they make a new best guess of the orientation to their target. This idea we propose of a ‘triggering response’ is essentially a modification of the ‘activation hypothesis' that has been discussed for migrating birds (e.g. [40]), whereby some types of information are not actually used for navigation, but rather elicit a response for an animal to make new decisions based on other navigational information. The slower travel speed as turtles travelled across the banks to their target might also have reflected opportunistic feeding and resting on this stage of migration, as seen during latter stages of green turtle migrations [15].

The navigational imperfection of turtles was evident right at the start of their oceanic migration, with their initial departure directions tending not to be target orientated, as is often the case with birds (e.g. [41]). Subsequent initial relatively straight-line oceanic routes suggest that turtles were unable to correct their course during these stages of short oceanic crossings, e.g. to account for any current deflections, in line with other studies (e.g. [15,36,42]). In some cases, it is known that the best navigation strategy can be to miss a target to one side [43]. For example, when travelling to a mainland coast, it may be better to miss the target well to one side so that an innate response, e.g. to turn right when encountering the coast, would always be the correct one. Similarly, missing the target to one side has been shown in desert ants returning to their burrow [44]. In the same way, we found that turtles heading to targets on the Great Chagos Bank tended to travel westwards of their target. Missing their targets in this way may be the best strategy to maximize the chances that the bank is encountered, as there was generally much more bank area westwards rather than eastwards of the targets. So, for example, if turtles missed their targets slightly to the east, they might be more likely to remain in oceanic water and miss the Great Chagos Bank entirely.

Around the world, relatively short migrations have been widely reported for hawksbill turtles [45]. They are thought to feed primarily on sponges that are widely distributed in shallow water in tropical and subtropical areas [21] and so may be located relatively close to nesting beaches. By contrast, migration distances are often much longer in green turtles which can travel many thousands of kilometres between nesting and foraging sites [45]. Green turtles feed predominantly on seagrass or macro-algae [20], which in tropical and subtropical areas may both be less ubiquitous than habitats with sponges. Hence, the often longer green turtle migrations may indicate that individuals are unaware of foraging sites close to their nesting beaches and so they return to more distant, familiar sites [46].

As with the hawksbill turtles tracked in our study, many sea turtle populations, across multiple species, breed on small islands or have their residential foraging sites far from land, making it likely that the searching in the final stages of migration occurs widely [15]. It might be thought that this need for island search might make island-nesting sites less popular than easier-to-find mainland sites. However, the isolation of breeding islands does not seem to limit the size of nesting populations. For example, Ascension Island in the central Atlantic is one of the largest green turtle rookeries in the world and yet also one of the most isolated, being greater than 1500 km from the nearest mainland coast [47]. The benefits of nesting on remote islands, which include a reduced risk of egg predation and historically less anthropogenic disturbance, are likely to outweigh the difficulties of finding such sites. The very circuitous routes that some individuals followed also suggest there might be occasions when turtles simply cannot find their target, be it an isolated foraging or breeding site. This suggestion is supported by some observations of turtles in unusual locations. For example, during the green turtle breeding season at Ascension Island, some turtle mating pairs are also seen on the island of St Helena, 1100 km to the south, even though that island has no nesting beaches [48]. The implication is that these mating turtles at St Helena were likely trying to find Ascension Island but became lost. Likewise prolonged searching behaviour has been documented in displacement experiments where turtles have been moved from isolated islands where they have been nesting and then released many tens of kilometres away [9,29,42,49]. In some cases, turtles were unable to locate their nesting island despite a protracted search, eventually giving up and returning to mainland foraging sites [49]. These occasional failures to locate an isolated target are supported by flipper tagging data that have revealed turtles may occasionally colonize new nesting areas and sometimes nest hundreds of kilometres or even greater than 1000 km from their original nesting area [50]. Such navigational imperfections may be permitted by the low ectothermic metabolic rate of sea turtles, which means that they have a long fasting endurance [51] allowing extended oceanic searches in the absence of food. Furthermore, these navigational imperfections may become increasingly important and might even be adaptive, in allowing new nesting sites to be established as climate change and associated sea-level rise threaten present-day nesting beaches. In the same way, it is possible that some turtles travelling to isolated foraging sites might sometimes fail to locate their target and instead move to new foraging sites. However, such instances have not been documented and so are presumably very rare [19].

In conclusion, the combined findings from satellite-tracked green turtles [15] and hawksbill turtles (this study) suggest that sea turtles locate isolated targets through a roughly target-oriented ocean crossing, open ocean course corrections and then localized search closer to the target. This conclusion is consistent with suggestions for laboratory trials with juvenile turtles showing the course resolution of the turtles' magnetic map [13]. As such, this combination of tracking free-living animals and laboratory trials helps solve a more than century-old riddle of how sea turtles complete long-distance migrations. Our findings are consistent with the suggestion that homing efficiency improves as the number of used cues is increased [52] and also reiterates the importance of considering current drift when examined the navigation performance of swimming animals [53].

## Data Availability

The raw turtle tracking data are in the electronic supplementary material, Information, table S1 [54].
